# P69 Computational drug-target interaction of antibacterial compounds from *Streptomyces chrestomyceticus* ADP4

**DOI:** 10.1093/jacamr/dlaf230.076

**Published:** 2025-12-04

**Authors:** Aakash Kumar, A K Dubey

**Affiliations:** Drug Discovery Lab, Department of Biological Sciences and Engineering, Netaji Subhas University of Technology, New Delhi, India; Drug Discovery Lab, Department of Biological Sciences and Engineering, Netaji Subhas University of Technology, New Delhi, India

## Abstract

**Objectives:**

To evaluate the drug-target interaction of the antibacterial compounds from the *Streptomyces chrestomyceticus* ADP4.

**Methods:**

*Staphylococcus aureus* is of the WHO priority pathogen, causative agent for multiple soft tissue and skin infections, gastroenteritis etc. making it a suitable candidate for investigation of the drug target based studies. Computational biology has emerged as promising, sustainable and efficient technique for shortlisting, screening and to predict drug target interaction and possible antibacterial mechanisms involved. The study involves molecular docking studies of compounds with the bacterial antibacterial targets of *S. aureus* which includes topoisomerase, metabolic enzymes such as malate dehydrogenase, dihydrofolate reductase etc. The protein structures were collected from protein data bank and homology models were constructed using templates with high sequence identity to get reliable 3D structure. The blind docking was performed using the PyRx software using the AutoDock Vina. Molecular dynamic (MD) simulation analysis was carried out using GROMACS tool. The drug-target complexes were screened and selected based on the threshold binding energy from molecular docking. The trajectory obtained from MD simulation were analysed for the time dependent interaction, conformational changes, stability, residue flexibility, compactness, solvent accessible surface area (SASA) of the drug- target complexes.

**Results:**

Molecular Docking analysis showed that the compounds interact and bind with target enzyme with intermediate binding affinity upto-7kcal/mol with decent stability and interaction in MD analysis. It further indicates the compounds as promising antibacterial agents with further scope of lead optimization for better antimicrobial potential.

**Conclusions:**

The study also highlights on the mode of action of the compounds by predicting the inhibition of the key metabolic enzymes and therefore metabolic pathways leading to bactericidal activity. This approach helps fast tracking the drug discovery initiatives time bound and early stage identification of the antimicrobial molecules.

